# Vanishing pachy-choroid in pachychoroid neovasculopathy under long-term anti-vascular endothelial growth factor therapy

**DOI:** 10.1186/s12886-021-02022-1

**Published:** 2021-06-30

**Authors:** Benedikt Schworm, Nikolaus Luft, Leonie F. Keidel, Thomas C. Kreutzer, Tina R. Herold, Siegfried G. Priglinger, Jakob Siedlecki

**Affiliations:** Department of Ophthalmology, Ludwig-Maximilians-University, Mathildenstrasse 8, 80336 Munich, Germany

**Keywords:** Pachychoroid, Pachychoroid neovasculopathy, Central serous chorioretinopathy, Choroidal neovascularization, Optical coherence tomography, Vascular endothelial growth factor, Ranibizumab

## Abstract

**Background:**

To investigate the diagnostic value of choroidal thickness in the definition of pachychoroid neovasculopathy (PNV), especially in eyes treated with anti-vascular endothelial growth factor (VEGF) therapy.

**Methods:**

Twenty-two consecutive eyes of 11 patients with uni- or bilateral PNV were analyzed. Anti-VEGF treatment was correlated with changes in choroidal thickness on enhanced depth imaging optical coherence tomography.

**Results:**

There were 14 eyes with PNV and 8 non-neovascular partner eyes. Mean age was 64.2 ± 4.0 (range: 60–72), total follow-up was 1.8 ± 0.4 (1–2) years. In PNV eyes, choroidal thickness at baseline was 400 ± 58 (269–485) μm. After two years and 13 anti-VEGF injections on average, a mean reduction of − 39 ± 10 (− 26 to − 56) % to final 241 ± 52 (162–327) μm was observed (*p* < 0.0001). Meanwhile, choroidal thickness in the partner eyes remained stable (*p* > 0.13 for all comparisons). A significant correlation of choroidal thinning and anti-VEGF injection rate was observed at year one (r = − 0.79; R^2^ = 0.63; *p* = 0.00073) and two (r = − 0.69; R^2^ = 0.48; *p* = 0.019). While 85.7% of PNV eyes exceeded a pachychoroid threshold of ≥350 μm at baseline, this figure dropped to 21.4% at year one and 0% at year two.

**Conclusion:**

In PNV, choroidal thickness significantly decreases with anti-VEGF therapy, resembling a “vanishing pachy-choroid”, and thus does not represent a valid long-term diagnostic criterium, especially when differentiating PNV from nAMD.

## Key messages

**What was known:** Pachychoroid neovasculopathy (PNV) is characterized by choroidal neovascularization above a thick choroid. **What is new:** In PNV, choroidal thickness decreases with increasing amounts of anti-VEGF injections. Thus, choroidal thickness becomes an unreliable diagnostic criterium in pre-treated eyes when differentiating PNV from neovascular AMD.

## Background

Pachychoroid disorders of the macula are frequently complicated by a choroidal neovascularization (CNV) [1–3]. These include *central serous chorioretinopathy (CSC),* which can develop *secondary CNV* in up to 25% of cases [1, 3], and *pachychoroid neovasculopathy* (PNV), which represents CNV atop areas of thickened choroid without prior evidence of CSC [4].

Recently, Hwang et al. [5] described a significant overlap of multimodal imaging findings in PNV and CSC complicated by CNV, and suggested that both might be variants of the same disease. Simultaneously, we have recently suggested the use of the term *PNV* as a general term for both PNV and CSC complicated by CNV to facilitate the distinction of neovascular pachychoroid disorders from non-neovacular (CSC, pachychoroid pigment epitheliopathy (PPE)) and neovascular pachychoroid disease with an aneurysmal phenotype (polypoidal choroidal vasculopathy/or aneurysmal type 1 CNV) [6].

Due to the high amount of cumulative damage required for CNV formation in pachychoroid disease, patients with PNV are usually older than patients with pachychoroid pigment epitheliopathy (PPE) or acute CSC [2]. Resultingly, PNV is often mistaken as, sometimes unresponsive, neovascular age-related macular degeneration (nAMD) [7], which can be aggravated by the CNV-induced disruption of retinochoroidal tissue, especially if the CNV is mimicking drusenoid pigment epithelium detachments [8].

Although both nAMD and PNV react well to anti-vascular growth factor (VEGF) therapy [9–11], a thorough discrimination between both entities is essential for further research endeavors targeting the very different underlying etiopathologies beyond anti-VEGF (drusen accumulation with age-related choroidal thinning vs. choroidal thickening and Haller vein dilation).

Based on the definition of pachychoroid disease, the presence of a thick choroid should be a prerequisite for its diagnosis [2]. On the other hand, choroidal thickness is known to decrease with age [12, 13], and moreover, might be influenced by disease duration, complications such as CNV and resulting disease-modifying intravitreal anti-VEGF therapy influencing choroidal permeability and CNV [14–16]. The following study was therefore designed [17] to investigate the validity of choroidal thickness in the definition of late-stage neovascular non-aneurysmatic pachychoroid disease, and its ability to discriminate between pachychoroid disease and nAMD.

## Methods

### Participants

For this retrospective cohort study, all eyes presenting with treatment-naïve CNV at the Ludwig Maximilians-University Munich, Germany, between January 2017 and May 2019 were screened for cases of PNV eligible for inclusion in this study, defined as: (i) Presence of a pachychoroid, defined as a) a subfoveal choroidal thickness ≥ 350 μm, or ≥ 250 μm with a previous pre-documented history of CSC, b) with the presence of pachyvessels, defined as dilated lumina of the Haller’s layer and c) an attenuation of the overlying choroidal structures; (ii) Presence of a CNV; (iii) absence of CNV aneurysms/polyps; (iv) anti-VEGF therapy with a follow-up of ≥1 year; (v) absence of classic drusen or reticular pseudodrusen on either eye; (vi) absence of anti-mineralocorticoid or photodynamic therapy during follow-up; (vii) absence of confounding comorbidities (diabetic retinopathy, hereditary retinal disease, diseases of the vitreoretinal interface, status after vitrectomy, optic media opacification impeding sufficient image quality). Unaffected partner eyes were included as a control group. Institutional review board approval was obtained for this retrospective chart review, and the study adhered to the tenets of the Declaration of Helsinki. At the moment of data analysis, ethic’s committee approval was not required for retrospective, non-interventional studies.

Epidemiological data was obtained from each patient, including age, gender, previous ocular comorbidities and procedures, date of first diagnosis of PNV, date of first anti-VEGF injection, number of anti-VEGF injections, and objective refraction-based Snellen chart visual acuity at baseline and at years 1 and 2, which was later converted to logMAR for analysis.

### Multimodal imaging

Multimodal imaging (all on Spectralis HRA + OCT, Heidelberg Engineering, Heidelberg, Germany) was performed after pupil dilation with topical tropicamide 1% and phenylephrine 2.5%. It included enhanced depth (EDI) spectral domain optical coherence tomography (SD-OCT) and near-infrared (NIR) confocal laser scanning ophthalmoscopy (CSLO) in every eye at each visit. OCT angiography was performed in every eye at baseline. Blue-autofluorescence (BAF) CSLO, fluorescein (FA) and/or indocyanine green (ICG) angiography and additional OCT angiography scans were performed at the investigator’s discretion. Only OCT images with a quality score of 20 and above were analyzed.

### Measurement of choroidal thickness

Choroidal measurements were obtained using the Heidelberg Eye Explorer (Heidelberg Engineering, Heidelberg, Germany) on enhance depth imaging OCT images in the 1:1 μm setting by two readers (JS, LK). In the case of disagreement, defined as a difference of > 10 μm [18, 19], a third senior reader was consulted (BS). Sub-foveal choroidal thickness (SFCT) was measured directly underneath the fovea from the outer portion of the retinal pigment epithelium to the sclerochoroidal interface. Two further choroidal thickness measurements were obtained in analogous fashion in a distance of 1500 μm nasally and temporally of the fovea.

### Anti-VEGF treatment

Treatment was primarily performed using a treat & extend regimen as described previously [20, 21]. In brief, each newly diagnosed CNV lesion was treated with an upload of three monthly injections of Ranibizumab (Novartis Pharma AG, Basel, Switzerland), after which the interval was extended or shortened by two weeks based on the absence or presence of new CNV activity. CNV activity was defined on OCT as (I) any new macular fluid, including SRF, (II) pigment epithelium detachment (PED) increasing central macular thickness > 50 μm, (III) new or increasing macular hemorrhage, or (IV) a decrease in visual acuity ≥1 Snellen chart line. Minimum and maximum interval between injections were 4 and 12 weeks, respectively. In the case of CNV reactivation, treatment was again started as in a newly diagnosed lesion. When patients did not consent with a treat & extend regimen, a pro-re-nata regimen was performed, using the same reactivation criteria as mentioned above.

### Statistical analysis

All data were gathered and analyzed in Microsoft Excel spreadsheets (Version 16.23 for Mac; Microsoft, Redmond, WA, USA). Statistical analysis was performed in SPSS Statistics 25 (IBM Germany GmbH, Ehningen, Germany). The level to indicate statistical significance was defined as *p* < 0.05. The Shapiro-Wilk and Kolmogorov-Smirnov tests were employed to test for normal distribution. Statistical analyses of intra-group differences were performed using the dependent two-tailed Student t-test and the Wilcoxon signed rank test. A repeated measures ANOVA test was used to compensate for multiple testing, if applicable. Pearson’s correlation coefficient was used to test associations of dependent and independent variables.

## Results

### Baseline demographics

Twenty-two eyes of 11 patients were included in the analysis. Detailed baseline parameters of the study cohort can be found in Table 1. In brief, 14 (63.6%) out of the 22 eyes had a diagnosis of PNV, and 8 eyes (36.4%) were partner eyes without signs of neovascularization. Mean age was 64.2 ± 4.0 (range: 60–72) years with a female to male ratio of 4 / 7 (36.4 / 63.6%). Total follow-up was 1.8 ± 0.4 (1–2) years. All eyes were available for the one-year analysis, and 77.2% were available at year two. In total, 10 of the 22 eyes (45.5%) had a history of CSC. In the anti-VEGF treatment group, these were 9 out of 14 eyes (64.3%). In the control group, one eye out of 8 had a history of CSC (12.5%), while 4 had PPE (50%).

**Table 1:** Baseline demographic data

No. of eyes (n)	22
PNV eyes	14
Right / Left	8 / 6
Partner eyes	8
Right / Left	3 / 5
No. of patients (n)	11
Gender (m/f)	7/4
Mean age (years)	64.2 ± 4.0 (range: 60–72)
Mean follow-up (y)	1.8 ± 0.4 (1.00–2.00)
Year 1	22 (100%)
Year 2	17 (77.2%)
Pachychoroid disease stage	
PNV eyes (*n* = 14)	
1 (PPE)	0
2 (CSC)	0
3 (PNV)	14 (100%)
4 (PAT1)	0
Partner eyes (*n* = 8)	
1 (PPE)	4 (50%)
2 (CSC)	1 (12.5%)
3 (PNV)	0
4 (PAT1)	0
Macular morphology	
PNV eyes	
Subretinal fluid	14 (100%)
Intraretinal fluid	0
Flat, irregular PED	14 (100%)
CNV	14 (100%)
Partner eyes	
Subretinal fluid	1
Intraretinal fluid	0
Flat, irregular PED	0
CNV	0
Mean Ranibizumab injections	
PNV eyes	
Year 1	7.0 ± 2.9 (2–11)
Year 2	6.5 ± 3.1 (1–10)
Partner Eyes	
Year 1	0
Year 2	0

### Macular morphology

At baseline, all eyes (100%) diagnosed with PNV had subretinal fluid and a flat, irregular PED forming a double-layer sign. The presence of a type 1 CNV within the flat PED was confirmed on OCT angiography in all eyes (100%). No eye showed intraretinal fluid or a type 2 CNV configuration at baseline.

In the 8 partner eyes, no case of flat irregular PED, CNV or intraretinal fluid was observed. Five eyes (62.5%) showed characteristics of the non-neovascular spectrum of pachychoroid disorders. In 4 (50.0%), pachychoroid pigment epitheliopathy (PPE) was found, while one eye (12.5%) displayed CSC with subretinal fluid at baseline, which resolved without treatment after 9 weeks of follow-up.

### Anti-VEGF treatment

In the first year, mean 7.0 ± 2.9 (2–11) injections were given. Due to disease inactivation after two injections, one patient expressed the wish to stop treatment on one eye (7.1%). All other patients received an upload of Ranibizumab (92.9%), after which three (23.1%) asked for a continuation of treatment using a pro-re-nata regimen. In the remaining 10 patients, a treat & extend regimen with mean 8.5 ± 1.6 (8–11) injections was performed. In the second year, 11 out of the 14 PNV eyes (78.6%) were treated with a mean of 6.5 ± 3.1 (1–10) injections. In these 11 eyes, a treat & extend regimen was used in 9 (81.8%), and a pro-re-nata regimen in 2 eyes (18.2%).

### Subfoveal, nasal and temporal choroidal thickness

**Fig. 1:**
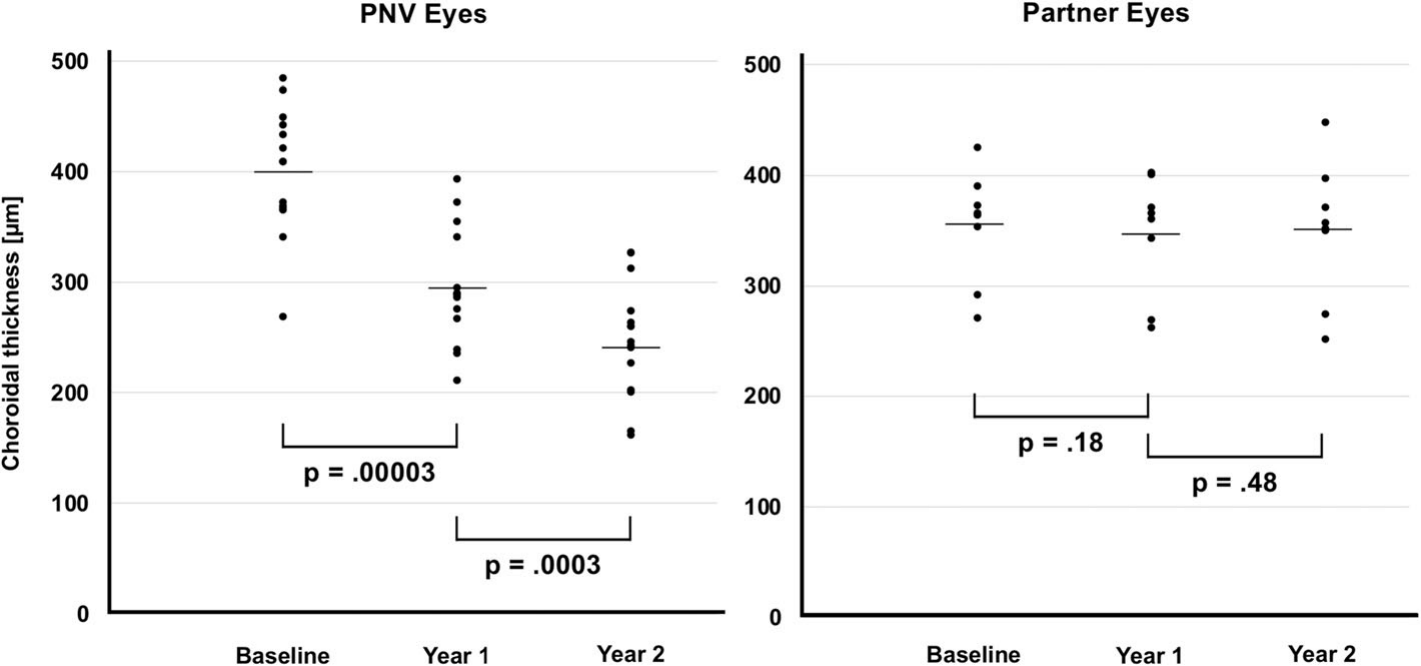
Changes in subfoveal choroidal thickness (SFCT) during follow-up. In PNV eyes, SFCT significantly decreased by 39% from baseline to end of follow-up, In partner eyes, SFCT remained unchanged during follow-up

Choroidal thickness during follow-up is presented in detail in Fig. 1 and Table 2. In PNV eyes, mean SFCT at baseline was 400 ± 58 (269–485) μm. At year one, it significantly decreased to 295 ± 53 (210–393) μm (− 25%; *p* < 0.0001). In year two, SFCT further decreased to 241 ± 52 (162–327) μm, resulting in an additional 20% reduction (*p* = 0.0003). Comparing baseline to end of follow-up at year two, a mean reduction of − 39 ± 10 (− 26 to − 56) % occurred. Mean nasal choroidal thickness (CT) at baseline was 312 ± 42 (243–368) μm. At year one, a decrease to 249 ± 47 (180–332) was observed (− 19%; *p* < 0.0018). In year two, nasal CT further decreased to 222 ± 40 (178–315) μm, resulting in an additional 12% reduction (*p* = 0.017). Comparing baseline to end of follow-up at year two, a mean reduction of − 27 ± 16 (− 52 to 1) % occurred. Mean temporal choroidal thickness (CT) at baseline was 324 ± 71 (174–404) μm. At year one, a decrease to 249 ± 51 (139–316) μm was observed (− 23%; *p* < 0.0001). At year two, temporal CT further decreased to 220 ± 46 (108–263) μm, resulting in an additional 11% reduction (*p* = 0.031). Comparing baseline to end of follow-up at year two, a mean reduction of − 29 ± 15 (− 56 to 2) % occurred.

**Table 2:** Choroidal thickness in PNV and partner eyes during follow-up

	Baseline choroidal thickness (μm)	Year 1 choroidal thickness (μm)	% reduction	p	Year 2 choroidal thickness (μm)	% reduction	p
**Eyes with PNV**							
subfoveal	400.3 ± 57.9 (269–485)	295.3 ± 52.8 (210–393)	− 0.25 ± 0.14 (− 0.50 to − 0.01)	.**00003**	241.4 ± 52.4 (162–327)	−0.20 ± 0.13 (− 0.38 to − 0.04)	**.0003**
nasal	311.9 ± 42.1 (243–386)	249.0 ± 47.2 (180–332)	− 0.19 ± 0.17 (− 0.49 to 0.07)	**.0018**	221.5 ± 40.2 (178–315)	−0.12 ± 0.16 (− 0.35 to 0.15)	**.017**
temporal	323.9 ± 71.1 (174–404)	248.6 ± 50.8 (139–316)	−0.23 ± 0.09 (− 0.38 to − 0.02)	**< 0.00001**	219.5 ± 45.8 (108–263)	−0.11 ± 0.15 (− 0.43 to 0.17)	**.031**
**Partner eyes**							
subfoveal	354.5 ± 50.3 (271–425)	347.5 ± 54.1 (262–403)	−0.02 ± 0.04 (− 0.10 to 0.03)	.19	350.5 ± 74.7 (252–449)	−0.01 ± 0.10 (− 0.14 to 0.15)	.48
nasal	264.1 ± 43.8 (183–310)	257.1 ± 55.7 (158–324)	−0.03 ± 0.13 (− 0.16 to 0.18)	.57	266.5 ± 55.1 (183–324)	0.03 ± 0.12 (− 0.13 to 0.23)	.50
temporal	301.4 ± 50.0 (220–384)	306.6 ± 56.7 (195–364)	0.02 ± 0.11 (− 0.12 to 0.16)	.67	262.3 ± 33.6 (200–296)	−0.07 ± 0.09 (− 0.17 to 0.07)	.13
		correlation with number of injections: r = − 0.79; R^2^ = 0.63; *p* = .00073			correlation with number of injections: r = − 0.69; R^2^ = 0.48; *p* = .019		

At baseline, the 8 partner eyes showed a mean SFCT of 355 ± 50 (271–450), a nasal CT of 264 ± 44 (183–310) and a temporal CT of 301 ± 50 (220–384) μm. At year one and two, mean SFCT, nasal and temporal CT remained unchanged (*p* > 0.13 for all comparisons).

### Correlation of choroidal thickness reduction and anti-VEGF injections

**Fig. 2:**
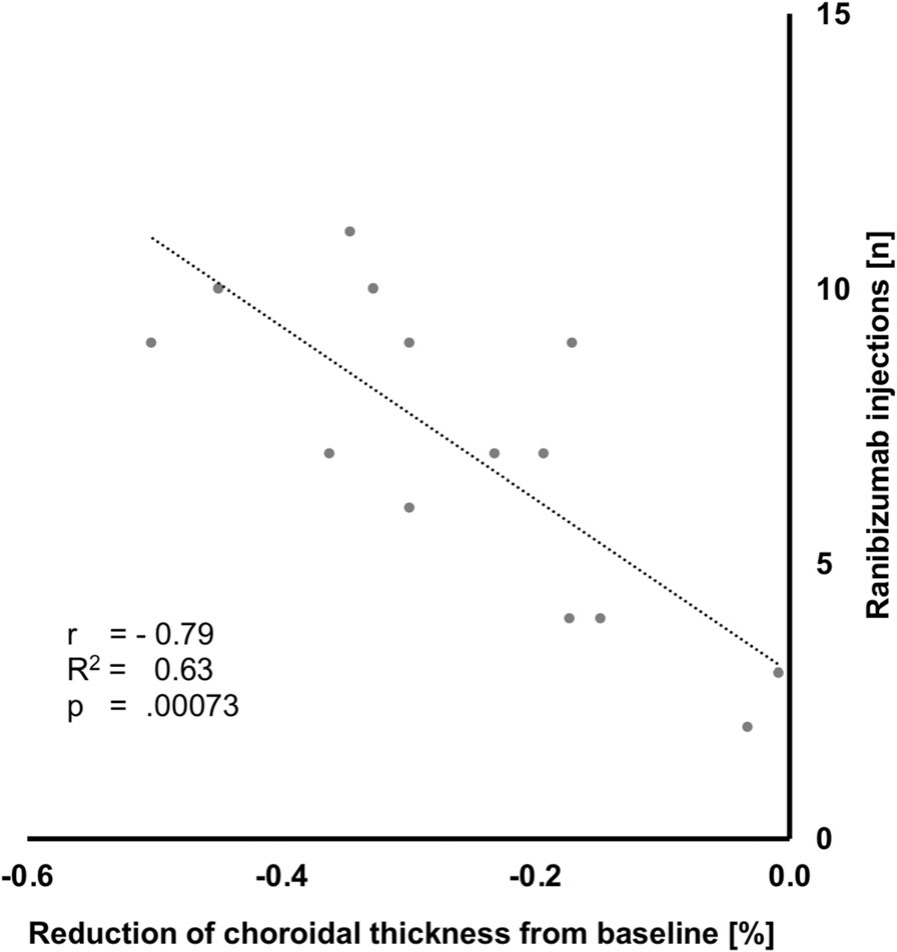
Correlation of subfoveal choroidal thinning and amount of anti-VEGF injections in the first year. A significant strong negative correlation was found (r = − 0.79; R^2^ = 0.63; *p* = .00073; Fig. 1). In year two, this correlation was maintained (r = − 0.69; R^2^ = 0.48; *p* = .019)

To test the hypothesis whether the absolute number of anti-VEGF injections correlated with the reduction in choroidal thickness, the Pearson correlation coefficient was calculated. In the first year, a significant strong negative correlation was found (r = − 0.79; R^2^ = 0.63; *p* = .00073; Fig. 2), which was also maintained in the second year (r = − 0.69; R^2^ = 0.48; *p* = .019).

### Choroidal thickness and pachychoroid threshold values

**Fig. 3:**
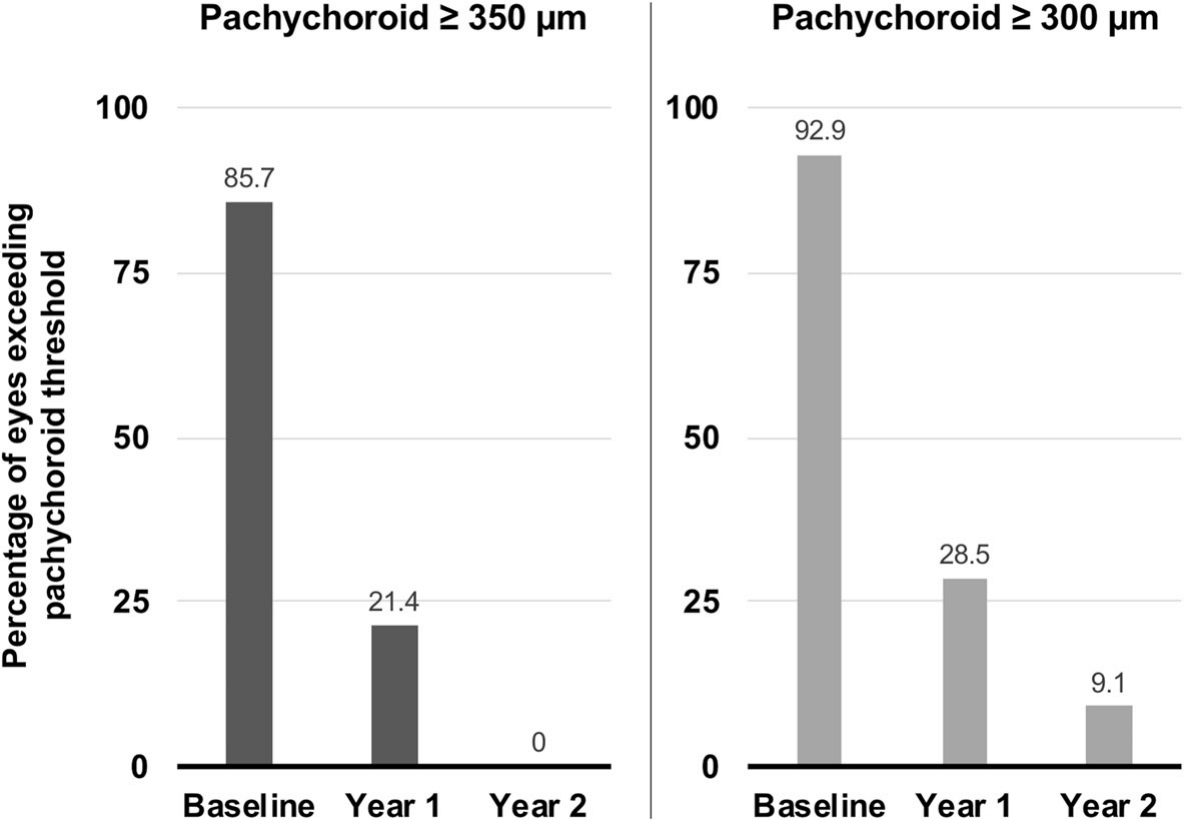
Percentage of PNV eyes fulfilling pachychoroid disease diagnostic thresholds at baseline, year one and two. At baseline, 12 out of the 14 PNV eyes (85.7%) exceeded a pachychoroid threshold of ≥350 μm. After anti-VEGF treatment, at year one, this figure dropped to 3 eyes (21.4%), and at year two, no eye fulfilled the threshold. Considering a pachychoroid threshold of ≥300 μm, 13 out of the 14 PNV eyes (92.9%) exceeded this value at baseline. After anti-VEGF treatment, this figure dropped to 4 eyes (28.5%) at year one, and one eye (9.1%) at year two

At baseline, 12 out of the 14 PNV eyes (85.7%) exceeded a pachychoroid threshold of ≥350 μm. After anti-VEGF treatment, at year one, this figure dropped to 3 eyes (21.4%; Fig. 3), and at year two, no eye fulfilled the pre-specified threshold.

Considering a pachychoroid threshold of ≥300 μm, 13 out of the 14 PNV eyes (92.9%) exceeded this value at baseline. After anti-VEGF treatment, this figure dropped to 4 eyes (28.5%; Fig. 3) at year one, and one eye (9.1%) at year two.

Of the 8 partner eyes, 6 (75%) showed a pachychoroid > 350 μm at baseline, which remained stable with 6 eyes (75%) at year one and 4 out of 6 eyes (66.7%) at year two. The exact same numbers apply for a pachychoroid threshold of > 300 μm.

### Visual acuity

In the PNV eyes, a mean visual acuity of 0.58 ± 0.39 (0.1–1.3) logMAR at baseline was maintained at year one with 0.54 ± 0.32 (0.1–1.0) logMAR and at year two with 0.68 ± 0.36 (0.1–1.3) logMAR (*p* = 0.63). In the partner eyes, the baseline visual acuity of 0.21 ± 0.24 (0.0–0.70) logMAR remained unchanged at year one with 0.28 ± 0.37 (0.0–1.0) logMAR and at year two with 0.22 ± 0.39 (0.0–1.0) logMAR (*p* = 0.88).

### Adverse events

No serious adverse events (no endophthalmitis, no retinal detachment, and no macular hemorrhage involving the fovea and requiring pneumatic displacement) were observed during the study period.

## Discussion

In the present study, we sought to test whether choroidal thickness represents a stable, reliable diagnostic criterium to discriminate between pachychoroid and non-pachychoroid neovascular disease, in this case pachychoroid neovasculopathy (PNV).

In accordance with the literature [2, 4, 11, 22], we found that the PNV eyes included in our study showed an above-average mean subfoveal choroidal thickness (SFCT) of 400 μm, which is well above the generally established threshold of > 350 μm considered to represent a pachychoroid [2]. As a new finding, we however describe that this “pachy-choroid” actually disappears with anti-VEGF therapy, and thus does not represent a reliable long-term diagnostic criterium. At two years, after mean 13 anti-VEGF injections, the PNV eyes included in this study showed a mean SFCT reduction by 39% to a final value of 241 μm, making them, without knowledge of the *status quo ante* at baseline, ineligible for a pachychoroid diagnosis based on above mentioned threshold. This resonates with recent findings on various treatment regimen for PNV [16, 23].

From a clinical perspective, the percentage of eyes presenting with a pachychoroid > 350 μm decreased from 86% at baseline to 21% at year one, and 0% at year two. While it is difficult to give a definite threshold above which choroidal thickness becomes pathological, Cheung et al. state in their major review of the pachychoroid spectrum that “a value exceeding 300 μm will mostly be regarded as pathological” [2]. This is mainly based on a series of older an newer studies, in which normal sub-foveal choroidal thickness was found to be around 300 μm on average (Margolis and Spaide [24] 287 μm, Manjunath et al. [25] 272 μm, Ikuno et al. [26]354 μm, Akhtar et al. [13] 307 μm). Applying this lower threshold of 300 μm to our study, 93% of eyes fulfilled this criterium at baseline – a figure which however also dropped to 29% at year one, and 9% at year two.

Strikingly, not all eyes included in this analysis showed a classic “pachychoroid” of > 350 μm at baseline – even though their “pachychoroid” origin was determined by pre-documented CSC in these eyes. Many factors have been shown to influence SFCT, mainly age [13] and axial length [27]. Akhtar et al. for example found a mean subfoveal thickness of 327 ± 68 μm for participants aged 12–18 years, as opposed to a mean 262 ± 72 μm in participants above 60 years [13]. In a study by Flores-Moreno et al. [27], choroidal thickness decreased by approximately 26 μm per every additional mm of axial length. In this context, our findings further establish anti-VEGF therapy as an important influence on choroidal thickness. In both year one and two, we saw a significant strong correlation of choroidal thinning and the amount of anti-VEGF injections applied. While these data already give a strong hint towards a possible connection, they are at the same time confirmed by the fact that the untreated partner eyes did not show any significant changes in choroidal thickness, though they largely also suffered from a pachychoroid and pachychoroid-related disease (pachychoroid pigment epitheliopathy, CSC without CNV). This difference between PNV and partner eyes, as well as the follow-up of just below two years should make the influence of aging as a confounding factor rather negligible.

Thinning of the choroid under anti-VEGF has been previously reported for diabetic macular edema [28], neovascular AMD [14], retinal vein occlusion [29] and myopic CNV [30], although some results are conflicting [31]. It is currently unclear which specific changes in the choroid contribute to its thinning during the course of intravitreal anti-VEGF therapy for PNV. Progresive damage to the choriocapillaris, and maybe even Sattler’s layer in advanced cases, which PNV certainly signifies [1, 2, 32], might hint at the pathologically dilated Haller’s layer to be the primary location of anti-VEGF action. Within Haller’s layer itself, decreases in luminal or stromal, or both departments may be responsible. Recent data analyzing choroidal changes in aging have found that both choroidal stromal and vessel volume decrease in a similar manner, maintaining the choroidal vascularity index (CVI) unaltered [33]. If pachychoroid disorders are to be understood as vascular congestion [34], and if the therapeutic effect of anti-VEGF should rely in partially alleviating this congestion, it might be intuitive to expect more luminal than stromal changes. Indeed, a recent study analyzing the effect of anti-VEGF on PCV (belonging to the same pathophysiological spectrum as PNV) found that the greatest vertical choroidal vessel diameter indicating choroidal lumina significantly decreased with anti-VEGF administration, and increased again prior to the reappearance of macular fluid on OCT. [35] These findings suggest that choroidal thickening in pachychoroid disorders might not only be secondary to dilated, congested vessels with increased diameter due to increased lumen, but also due to leakage from the choroidal lumina into the stroma. With this hypothesis, the thinning effects of anti-VEGF on the choroid might be most intuitively explained by a reduction of choroidal leakage from pachyvessels, and thus less stroma swollen by leaked fluid. Due to this dual action, eyes with PNV might require less injections than type 1 CNV in AMD, and visual acuity results might be better as recently suggested [36]. On the contrary, photodynamic therapy might be beneficial if anti-VEGF manages CNV, but fails to normalize choroidal congestion [37].

Our data directly translate into possible recommendations as how to diagnose PNV. Choroidal thickness should be referenced at baseline, and not used as a diagnostic criterium after commencing anti-VEGF treatment. Moreover, our data also indirectly translate into a better distinction of neovascular pachychoroid disease and age-related macular degeneration (nAMD) [38, 39]. It is conceivable that a certain percentage of presumed neovascular AMD, in reality, represents PNV. A relatively high incidence of misdiagnosis might be aggravated by the fact that choroidal thickness does not serve as a good differentiator between both entities in the chronic, anti-VEGF treatment phase. It is these chronically ill eyes, however, in which “AMD non-response” is mostly evaluated – and where PNV can serve as an important differential diagnosis bearing treatment decisions regarding on disease-specific biomarkers. While persistent subretinal fluid, for example, can potentially be tolerated in nAMD [40], it will inevitably lead to progressive foveal thinning in pachychoroid disease [41], for which several additional treatment options are under current investigation, including mineralocorticoid receptor antagonists [42, 43], photodynamic therapy, or non-damaging laser treatments [44]. These options should not be withheld to a patient based on a false diagnosis of presumed nAMD. For these reasons, other biomarkers of pachychoroid disease should be first choice apart from sole choroidal thickness when the eye in question presents with a history of anti-VEGF treatment. These biomarkers include, e.g., the presence of dilated Haller veins, called pachyvessels, an increased choroidal vascularity index, and choroidal hyperpermeability [2, 45].

Certain limitations of our study can be found. First of all, our sample size as well as follow-up are limited. Moreover, we cannot give a detailed analysis of choroidal perfusion changes under anti-VEGF therapy as we lack consistent long-term OCT angiography data. Moreover, 14% of PNV eyes included in this study already presented with SFCT below the 350 μm pachychoroid threshold before commencing anti-VEGF therapy; in these eyes, however, the diagnosis of PNV was confirmed due to a pre-documented history of chronic CSC. Another statistical limitation lies within the inclusion of both eyes of every patient, which indicates that the data of the treatment and control group are not statistically independent. This limitation however cannot be overcome as this study was specifically designed to include the partner eye as an intra-individual control which undergoes the same systemic factors influencing SFCT as in the treated eye (e.g. blood pressure, systemic glucocorticoid levels, aging).

In conclusion, this study demonstrates that choroidal thickness in PNV significantly decreases with anti-VEGF therapy, resembling a “vanishing pachy-choroid”. Thus, choroidal thickness does not represent a valid long-term diagnostic criterium and cannot be used as a safe differentiator from nAMD in these cases [46, 47]. Further analyses of pachychoroid biomarkers beyond sole thickness are warranted, e.g. towards choroidal vascularity index and choroidal stroma-to-vessel volume ratio, which might represent more stable, anti-VEGF independent biomarkers in the diagnosis of neovascular and non-neovascular pachychoroid disease [46].

## Data Availability

Data will be made available upon reasonable request.

## Abbreviations

BAF: blue autofluorescence
CNV: Choroidal Neovascularization
CSC: Central serous chorioretinopathy
CSLO: confocal scanning laser ophthalmoscopy
CT: Choroidal Thickness
EDI: Enhanced depth imaging
FA: Fluorescein angiography
ICGA: Indocyanine green angiography
nAMD: Neovascular age-related macular Degeneration
NIR: Near-infrared reflectance
OCT: Optical coherence tomography
PNV: Pachychoroid neovasculopathy
SFCT: Sub-foveal choroidal thickness
VEGF: Vascular endothelial growth factor
